# Changes in sulphur content, uptake, internal efficiency index, harvest index and N:S ratio in pea plants from the four-leaf stage to full maturity

**DOI:** 10.1186/s12870-026-09183-5

**Published:** 2026-06-08

**Authors:** Andrzej Wysokinski, Beata Wiśniewska-Kadżajan, Izabela Łozak

**Affiliations:** Faculty of Agricultural Sciences, Institute of Agriculture and Horticulture, University of Siedlce, Konarskiego 2 Street, 08110 Siedlce, Poland

**Keywords:** N:S ratio, Pea development stages, Pea aerial part, Pea roots, Pea seeds, *Pisum sativum* L., Sulphur content, Sulphur harvest index, Sulphur internal efficiency index, Sulphur uptake

## Abstract

**Background:**

Sulphur is an important macronutrient for legume plants because it influences the formation and functioning of root nodules, the activity of enzymes reducing molecular nitrogen to available forms for plants, the formation of storage proteins, and in effect, ultimately the yield and quality of the crop. Sulphur should be adequately available for these plants during the entire growing period, because it is poorly redistributed from older organs to younger ones. Therefore it is appropriate to determine suitable parameters illustrating sulphur management by the pea plant throughout the growing period and accumulation in various organs.

**Method:**

In a two-year study, we determined the sulphur content (Sc), sulphur uptake (Sup), sulphur internal efficiency index (SIE), sulphur harvest index (SHI), and N: S ratio in six development stages of pea according to Biologische Bundesanstalt, Bundessortenamt und Chemische Industrie (BBCH) scale: BBCH 14, BBCH 33, BBCH 55, BBCH 65, BBCH 75 and BBCH 90. Analyses were performed for the root and aerial part, and at the fully ripe stage, for seeds as well. The plants were fertilized with sulphur and nitrogen before sowing at rates of 34 kg·ha^− 1^ S and 30 kg·ha^− 1^ N, both in the form of ammonium sulphate (NH_4_)_2_SO_4_.

**Results:**

In most cases, the pea parameters were influenced by both the development stage and the year of the study, but not all of them were significantly influenced by the cultivar. The values of the measured parameters for the whole mass of peas ranged: Sc from 2.70 to 5.02 g S·kg^− 1^ dw, N:S from 8.5 to 11.5, Sup from 1.19 to 18.45 kg S ·ha^− 1^, SIE from 205 to 305, and SHI calculated for whole aerial mass from 0.49 to 0.98 (0.45 calculated for seeds in 90 BBCH). Sc, Sup and SHI calculated for whole pea plants increased between BBCH 14 and BBCH 33, while the N: S ratio and SIE decreased. Sc decreased from BBCH 33 stage to BBCH 75, while Sup, SIE and SHI increased, and the N: S ratio did not change significantly. Between BBCH 75 and BBCH 90, there were no significant changes in any of the parameters of sulphur management.

**Conclusion:**

All tested parameters of sulphur management indicated adequate supply of sulphur to the pea plants throughout the growing season.

## Introduction

Sulphur is a macronutrient involved in determining the quantity and quality of protein in plants [1, 2]. Sulphur deficiency in plants inhibits N and S metabolism levels [3], reduces the amount of sulphur-containing amino acids, while an increase in the sulphur level increases their content [4]. According to the author cited, sulphur deficiency also increases the content of non-protein nitrogen while reducing protein content. Therefore, inadequate supply of this macronutrient to plants reduces crop quality. Sulphur application increases both crop yield and the concentration of protein [5–7]. Nitrogen application is less effective in conditions of limited sulphur availability, whereas good sulphur availability increases nitrogen utilization from fertilizers [8]. Sulphur deficiency can reduce nitrogen use efficiency [9]. Moreover, sulphur is a component of numerous compounds, such as amino acids, vitamins, coenzymes, the thioredoxin system, lipoic acids, and glucosinolates, which directly or indirectly mitigate the adverse effects of biotic and abiotic stressors in plants, and also act as antioxidants [10–12].

Sulphur plays a special role in legume crops, both in the biological nitrogen reduction process and in protein formation [13–16]. The nitrogen reduction process involves the enzyme nitrogenase, of which sulphur is an important component [17–22]. A key element of nitrogenase reactivity is the active site cofactor, a complex metallocluster [19, 20]. This metallofactor contains three ‘belt sulphides’ (MoFe7S9C), and sulphur atoms form bridges between iron atoms and molybdenum atoms. The last (ninth) sulphur atom is inserted into the cluster at a late stage of its biosynthesis, which is essential to activate the enzyme. A dynamic in-and-out of belt-sulphurs takes place during the catalysis reaction [18]. Good supply of sulphur to legumes enables the normal development of nodule and functioning of rhizobia, leading to efficient binding of atmospheric nitrogen [23–25]. In contrast, sulphur deficiency limits symbiotic nitrogen binding, leading to N deficiency in the plant [26]. In effect, limited nitrogen availability reduces the amount of biomass produced. Low sulphur availability reduces the number and weight of root nodules [25–27], as well as their content of leghaemoglobin and ferredoxin [25]. In pot experiments, in the period up to 60 days after sowing of pea seeds, the dry weight of root nodules following sulphur application (200 mg S per 10-litre pot filled with perlite) was already several times as high as in the pots without sulphur application [26]. Good supply of sulphur and nitrogen to soybeans improves the quality of the protein produced [16], which is rich in sulphur-containing amino acids: methionine and cysteine [28, 29]. In the case of field pea, sulphur deficiency reduces the synthesis and level of sulphur-containing storage proteins (albumin and legumin), while at the same time increasing synthesis of storage proteins poor in cysteine and methionine [30, 31].

In the past, plants were often well-supplied with sulphur due to high soil resources, as well as atmospheric deposition [32–34]. However, due to the substantial decrease in the influx of sulphur to the atmosphere and from there to the soil, cases of sulphur deficiency in crops have appeared [1]. In these conditions, fertilization with sulphur has become important.

In legume plants, including pea, mineral fertilization is usually limited to pre-sowing application. This is justified by the need to ensure adequate availability of macro- and micronutrients for plants and rhizobia in the early stages of growth and root nodule formation, as well as for the activity of enzymes involved in biological reduction of molecular nitrogen. While the initial amount of nitrogen applied should ensure its availability until efficient symbiosis develops between the plant and bacteroids, the amount of other macro- and micronutrients applied, including sulphur, should be sufficient for the entire growing period. Sulphur is often applied in sulphate forms (-SO_4_) [35, 36], which, unfortunately, are easily leached from soil [4, 37]. Therefore, application in this form before sowing may not guarantee adequate sulphur availability for the duration of the growing period, especially in the final stages. This is important because sulphur is poorly redistributed from old plant organs to young ones [2]. Sulphur fertilization alone may not be an effective way to ensure its availability to plants, because optimal sulfur uptake, distribution, and assimilation depend on precise synchronization with plant development phases [34]. Throughout the growing season, the Sc in plant tissues, its Sup, the N: S ratio, the SIE index, and the SHI index can be parameters illustrating the availability of sulphur for plants, which is essential for producing yield, the transformation of reduced nitrogen into protein, and the redistribution of sulphur in the plant. Scherer and Lange [27] demonstrated that sulphur deficiency in legumes reduces protein synthesis in the seeds more than biomass growth, which makes the N: S ratio a better diagnostic than Sc alone.

The *Pisum sativum* L. species was chosen for this study because it is one of the most important crops in the world. It is a multipurpose plant: it is used for animal feed and human food, improves soil properties [38–41], and can be visually attractive in the landscape. Area harvested (ha), yield (kg⋅ha^− 1^) and production (tons) are, respectively: Cow peas (dry) 15,783,942 ha, 640 kg⋅ha^− 1^, 10102198.5 tons; Peas (dry) 7,358,769 ha, 1924 kg⋅ha^− 1^, 14157952.1 tons; Peas (green) 2,671,241 ha, 8135.6 kg⋅ha^− 1^, 21732165.8 tons; and Pigeon peas (dry) 5,283,797 ha, 881.6 kg⋅ha^− 1^, 4657959.1 tons [42].

Taking into account the lack of such current data in pea cultivation, the study was undertaken to determine:

1. the changes in Sc in plant tissues, Sup, and N: S ratio from the four-leaf stage to full maturity of multi-purpose and fodder pea cultivars, fertilized before sowing with sulphur and nitrogen in the form of ammonium sulphate.
2. the value of SIE and the SHI indexes in the same growth periods of both pea varieties.

The research undertaken is consistent with the Sustainable Development Goals (SDGs) of “Zero Hunger” and “Responsible Consumption and Production.” Adequate sulphur availability for legumes ensures the production of high-quality feed and food, increases the biological value of protein, and ultimately reduces qualitative malnutrition. Determining changes in the SIE index throughout the plant’s life cycle will allow us to understand how effectively peas convert absorbed sulphur into yield, while the SHI index will determine the efficiency of sulphur translocation to seeds and the amount of sulphur remaining in crop residues, which is crucial in a circular economy. Ultimately, it will be possible to assess the validity of the adopted sulphur fertilization method, as well as the need for adjustments and reassessment in subsequent experiments.

The adopted research hypothesis assumed:

1. In the initial growth stages, peas will achieve the highest Sc values, while in the phases of intensive biomass growth, Sup dynamics will be the highest, while the SIE and SHI indexes will increase with the growth and development of the test plant.
2. Pea varieties with different uses (general-purpose and forage) will demonstrate different sulfur management strategies, as reflected by the Sc, Sup, N:S ratio, SIE, and SHI indexes.
3. Conditions variabiliy during the years of the study will affect the variation in Sc, Sup, N:S ratio, SIE, and SHI values.

## Materials and methods

### Experiment design

The experiment was carried out in 2015 and 2016 in Siedlce city, eastern Poland (52°10′ N, 22°17′ E). The experiment was two-factorial, set up in a randomized block design in three replications. The first factor was two different pea cultivars: 1)‘Batuta’, a multi-purpose cultivar, 2)‘Milwa’, a fodder cultivar. The second factor was six (6) harvest dates in different growth phases of the tested plant (according to the BBCH scale): (1) BBCH 14, 4-leaf stage; (2) BBCH 33, 3-internode stage; (3) BBCH 55, stage when the first single buds are visible outside the leaves - abbrev. “first bud”; (4) BBCH 65, full flowering stage - abbrev. “flowering”; (5) BBCH 75, when 50% of pods are of typical length - abbrev. “50% pods”; and (6) BBCH 90, full maturity - abbrev. “maturity”. Each experimental plot had an area of 1 m^2^ and was located in a field of test cultivated plant. These plots were randomly distributed throughout the pea field. The total number of plots in each year of the experiment was 36: 2 cultivars, 6 harvesting dates, number of replications *n* = 3.

Pea plants (*Pisum sativum* L.) were grown in conventional soil cultivation system. Winter rye was the forecrop in both years of study. Pre-winter plowing was performed in the fall. Mineral fertilizers were applied in the spring, followed by tillage with a tiller and hand sowing of the seeds. Pea were sown by hand on April 08 of both years in the amount of 110 seeds per 1 m^2^.

The experimental material comprised certified seeds of two field pea (*Pisum sativum* L.) cultivars: ‘Milwa’ (breeder: Hodowla Roślin Smolice Sp. z o.o.) and ‘Batuta’ (breeder: Danko Hodowla Roślin Sp. z o.o.). The seeds were obtained in 2015 and 2016 from seed company “Plon”, Siedlce, Poland. Both cultivars are registered in the Polish National List of the Research Centre for Cultivar Testing (COBORU). The experiments were conducted in accordance with institutional and national guidelines for plant research. No specific permits were required for the use of these commercial cultivars, as they are widely available for agricultural and research purposes in the European Union.

Fertilization was carried out immediately before sowing the seeds. The following was used: (a) sulphur in the amount corresponding to the application of 34 kg·ha^-1^ S into the soil, i.e., 3.4 g·m^-2^ S, (b) nitrogen in an amount corresponding to the application of 30 kg·ha^-1^ N into the soil, i.e., 3 g·m^-2^ N, both (S and N) in the form of ammonium sulphate (NH_4_)_2_SO_4_, (c) potassium (K) in the amount corresponding to the application of 100 kg·ha^-1^ K into the soil, i.e., 10 g·m^-2^ K, in the potassium chloride (KCl) form. Phosphorus (P) was not applied, because the soil was shown to have very high content of this macronutrient in available forms (Table 1).

**Table 1:** Some properties of soil in the layer 0–0.25 m before foundation of experiment in 2015 and 2016

Soil properties	Units	Years	
		2015	2016
pH_1 mol·dm−3 KCl_	-	6.6	6.5
C_total_	g·kg^− 1^	34.2	23.5
N_total_		2.10	1.45
S_total_		0.364	0.350
S_0.03 mol·dm−3 CH3COOH_	mg·kg^− 1^	15.8	16.2
P_Egnera−Rhiema_		309.0	301.0
K_Egnera−Rhiema_		86.0	111.0

Before sowing, the pea seeds were inoculated pea symbiotic bacteria *Rhizobium leguminosarum*. No herbicides were used. Weeds were removed by hand.Some properties of the soil - Luvisols (LV) - are given in Table 1.

Before the experiment in the soils the following were determined:

- pH value in mol⋅dm^-3^ KCl - potentiometrically;
- Total N and carbon (C) - on a CHN autoanalyzer with IDC detector, Series II 2400, Perkin-Elmer, Valencia, CA, USA;
- P and K available for plants – by Egner-Riehm method, and determination of these elements concentration in obtained solution by Inductively Coupled Plasma Atomic Emission Spectroscopy (ICP-AES) method on spectrometer Optima 3200 RL, Perkin-Elmer, Waltham, USA;
- Total S - after wet mineralization in a mixture of concentrated hydrochloric acid (HCl) and nitric acid (HNO_3_) (3:1 ratio) using the ICP-AES spectrometer (as above);
- S in form available for plant by extraction in solution 0.03 mol·dm^-3^ acetic acid (CH_3_COOH) and determination of the S concentration in this solution by ICP-AES method (as above).

Whole pea plants – aerial and underground parts - were harvested manually at the specified growth phases by digging them out of the soil with a spade to a depth of 0.25 m. All plants were harvested separately from each 1 m^2^ plot. The plants were harvested in the first year of the experiment on dates: 08th May, 22th May, 05th June, 19th June, 27th June, and 28th July, while in the second year on days: 09th May, 23th May, 04th June, 15th June, 25th June, and 26th July.

### Laboratory analyses

Pea plants from each plot were counted (Table 2). Plants harvested at 14–75 BBCH phases were divided for roots and aboveground part from each plot, and at 90 BBCH phase pea seeds were separated additionally. Hereafter, in all stages of pea growth, all aboveground parts of the plant except for the seeds separated in maturity stage are referred to as the aerial part. The aerial part included all the aboveground organs of peas: stems, leaves, flowers, pods and stripped pods, respectively. The roots were washed with distilled water after harvest. The separated parts were dried at 70 °C [43] and ground. All samples of plant material were subjected to dry mineralization (ashing) at 450 °C. Obtained ash were dissolved in 6 mol·dm^-3^ HCl, which was subsequently evaporated. Residue after evaporation were transferred to volumetric flasks in 1% HCl solution. The content of sulphur in this solution was determined using the ICP-AES method (as above).

**Table 2:** Number of pea plants per 1 m^2^

Years of study	Cultivars	Growth stages					
		Four-Leaf	Three-internode	First bud	Flowering	50% Pods	Maturity
2015	Milwa	105.0	103.3	103.7	95.7	97.7	97.3
	Batuta	98.3	98.3	100.7	97.3	99.3	96.0
2016	Milwa	97.0	99.0	99.3	95.7	97.3	95.3
	Batuta	95.7	100.3	98.3	94.3	95.0	93.7

All analyses were performed in triplicate. STD GEOCHEM CUSTOM 4 standard reference solutions (PE #: N9307113) were used in the analytical process.

### Weather conditions

Analysis of the weather conditions during the growing season of pea showed that the temperature and rainfall were varied in the study years (Tables 3 and 4).

**Table 3:** Monthly air temperatures and rainfall in 2015–2016, (Institute of Meteorology and Water Management, National Research Institute in Warsaw)

Months	Average monthly temperatures in experimental years, °C			Total Monthly Rainfall in experimental years, mm		
	2015	2016	Long-Term Mean 1981–2014	2015	2016	Long-Term Mean 1981–2014
March	4.8	3.3	2.0	53.1	46.4	29.6
April	8.2	8.9	8.1	30.0	50.2	33.4
May	12.3	14.6	13.6	100.2	35.5	60.3
June	16.5	18.1	16.3	43.3	55.6	72.9
July	18.7	19.0	18.5	62.6	126.8	67.6
Average/Sum March-July	12.1	12.8	11.7	289.2	314.5	263.8
Average/Sum April-July	13.9	15.1	14.1	236.1	268.1	234.2

**Table 4:** Values of the Selyaninov hydrothermal index (k) during the vegetation periods of pea and spring barley and moisture characteristics (wm) of individual months

Month	Year			
	2015		2016	
	k	wm	k	wm
April	1.2	md	1.9	mw
May	2.6	vw	0.8	d
June	0.9	d	1.0	d
July	1.1	md	2.2	w

*k* ≤ 0.4—extremely dry (ed); 0.4 < *k* ≤ 0.7—very dry (vd); 0.7 < *k* ≤ 1.0—dry (d); 1.0 < *k* ≤ 1.3—moderately dry (md); 1.3 < *k* ≤ 1.6—optimum (o); 1.6 < *k* ≤ 2.0—moderately wet (mw); 2.0 < *k* ≤ 2.5—wet (w); 2.5 < *k* ≤ 3.0—very wet (vw); *k* > 3.0—extremely wet (ew)

Selyaninov’s hydrothermal index [44] indicates that, in 2015: April and July were moderately dry, May was very wet, and June was dry, but in 2016: April was moderately wet, May and June were dry, and July was wet.

### Calculations and statistical analysis

1. Accumulation (uptake) of sulphur by pea:

$$\:Sup=\frac{dw\cdot\:Sc}{1000}$$

Where:

Sup – sulphur accumulation (uptake) in pea’s separated parts [kg S·ha^-1^];

dw – dry weight [kg·ha^-1^] of separated pea’s part in the studied development phase [45];

Sc – sulphur content in the separated pea’s part in the studied development phase [g S·kg^-1^ dw];

Total accumulation (uptake) of sulphur in pea was calculated by summarising its uptake by all separated parts.

2) Nitrogen and sulphur ratio (N: S) was calculated by dividing N content [45] by S content in the separated parts of the pea and its whole mass.
3) Sulphur Internal Efficiency index (SIE):

$$\:SIE=\frac{dw}{Sup}$$

Where:

dw – dry weight [kg·ha^-1^] of the separated parts of pea and its whole mass [45];

Sup – amount (uptake) of sulphur accumulated in separated parts of the pea and in its whole mass [kg S·ha^-1^].

4) Sulphur harvest index (SHI):

$$\:SHI=\frac{Sup\_G}{Sup\_W}$$

Where:

Sup_G – sulphur accumulation (uptake) in aerial crop residues or seeds yield of pea, respectively [kg S·ha^-1^];

Sup_W – sum of sulphur accumulation (uptake) in pea roots, aerial crop residues and seeds (seeds only at 90 BBCH phase) – whole pea plant [kg S·ha^-1^].

Analysis of variance of the results was performed using an Microsoft Excel spread sheet. The effect of the development phase on studied parameters of pea was determined using three-factor analysis of variance and Fisher’s F-test. The effect of pea variety and years of study on studied parameters was calculated for data obtained at 90 BBCH using two-factor variance analysis. To test the significance of differences between mean values, least significant difference (LSD 0.05) values with *p* ≤ 0.05 were calculated by Tukey’s test.

Three-factor analysis of variance were performed according to the following model:

$$\begin{aligned}{\mathrm{y}}_{\mathrm{i}\mathrm{j}\mathrm{l}\mathrm{p}}\:&=\:\mathrm{m}\:+\:{\mathrm{a}}_{\mathrm{i}}\:+\:{\mathrm{b}}_{\mathrm{j}}\:+\:{\mathrm{c}}_{\mathrm{l}}\:+\:{\mathrm{a}\mathrm{b}}_{\mathrm{i}\mathrm{j}}\:\\&+\:{\mathrm{a}\mathrm{c}}_{\mathrm{i}\mathrm{l}}\:+\:{\mathrm{b}\mathrm{c}}_{\mathrm{j}\mathrm{l}}+\:{\mathrm{a}\mathrm{b}\mathrm{c}}_{\mathrm{i}\mathrm{j}\mathrm{l}}\:+\:{\mathrm{e}}_{\mathrm{i}\mathrm{j}\mathrm{l}}\end{aligned}$$

Where:

y_ijlp_ - the value of the examined characteristic.

m - population average.

a_i_ - the effect of pea development phase.

b_j_ - the effect of pea variety.

c_l_ - the effect of year.

e_ijl_ - the random error (numbers).

Two-factor analysis of variance were performed according to the following model:

$$\:{\mathrm{y}}_{\mathrm{i}\mathrm{j}\mathrm{p}}\:=\:\mathrm{m}\:+\:{\mathrm{a}}_{\mathrm{i}}\:+\:{\mathrm{b}}_{\mathrm{j}}\:+\:{\mathrm{a}\mathrm{b}}_{\mathrm{i}\mathrm{j}}\:+\:{\mathrm{e}}_{\mathrm{i}\mathrm{j}}$$

Where:

y_ijp_ - the value of the examined characteristic.

m - population average.

a_i_ - the effect of variety of pea.

b_j_ - the effect of years of study.

e_ij_ - the random error (numbers).

For selected pea’s biomass parameters, Pearson’s linear correlation coefficient was calculated using Statistica 13.1 PL software (StatSoft Inc., Tulsa, OK, USA).

## Results

### Sulphur content

Sc in the separated plant parts and on average in the whole plants changed significantly during the stages of pea development (Table 5). Sc in the roots and aerial part, as well as on average in the whole plant, increased from the four-leaf stage to the three-internode stage. Beginning with the three-internode stage, its content in the roots and on average in the whole plant decreased up to the 50% of pods stage, and in the aerial part, until full maturity. Sc in the roots and on average in the whole plant was similar in the last two stages of development. At full maturity, the content of this macronutrient was highest in the seeds.

**Table 5:** The content of sulphur in pea, mean values for varieties and years, ± standard deviation (SD), g S·kg^− 1^ dry weight (dw)

Parts of pea plants	Growth stages						LSD_0.05_
	Four-Leaf	Three-internode	First bud	Flowering	50% Pods	Maturity	
Seeds						3.24 (± 0.23)	-
Aerial part	3.12c (± 0.26)	4.03e (± 0.58)	3.59d (± 0.49)	3.02bc (± 0.23)	2.81b (± 0.36)	2.40a (± 0.29)	0.27
Roots	4.32b (± 1.10)	8.28d (± 2.31)	4.97c (± 1.44)	4.12b (± 0.64)	2.64a (± 0.31)	2.22a (± 0.35)	0.53
Weighted averages	3.55c (± 0.35)	5.02d (± 0.88)	3.71c (± 0.53)	3.08b (± 0.24)	2.81a (± 0.35)	2.70a (± 0.21)	0.25

a, b,c, d – means for investigated factors with different lowercase letters in the rows are significantly different. *n*.*s*. Not significantly differ

**Fig. 1:**
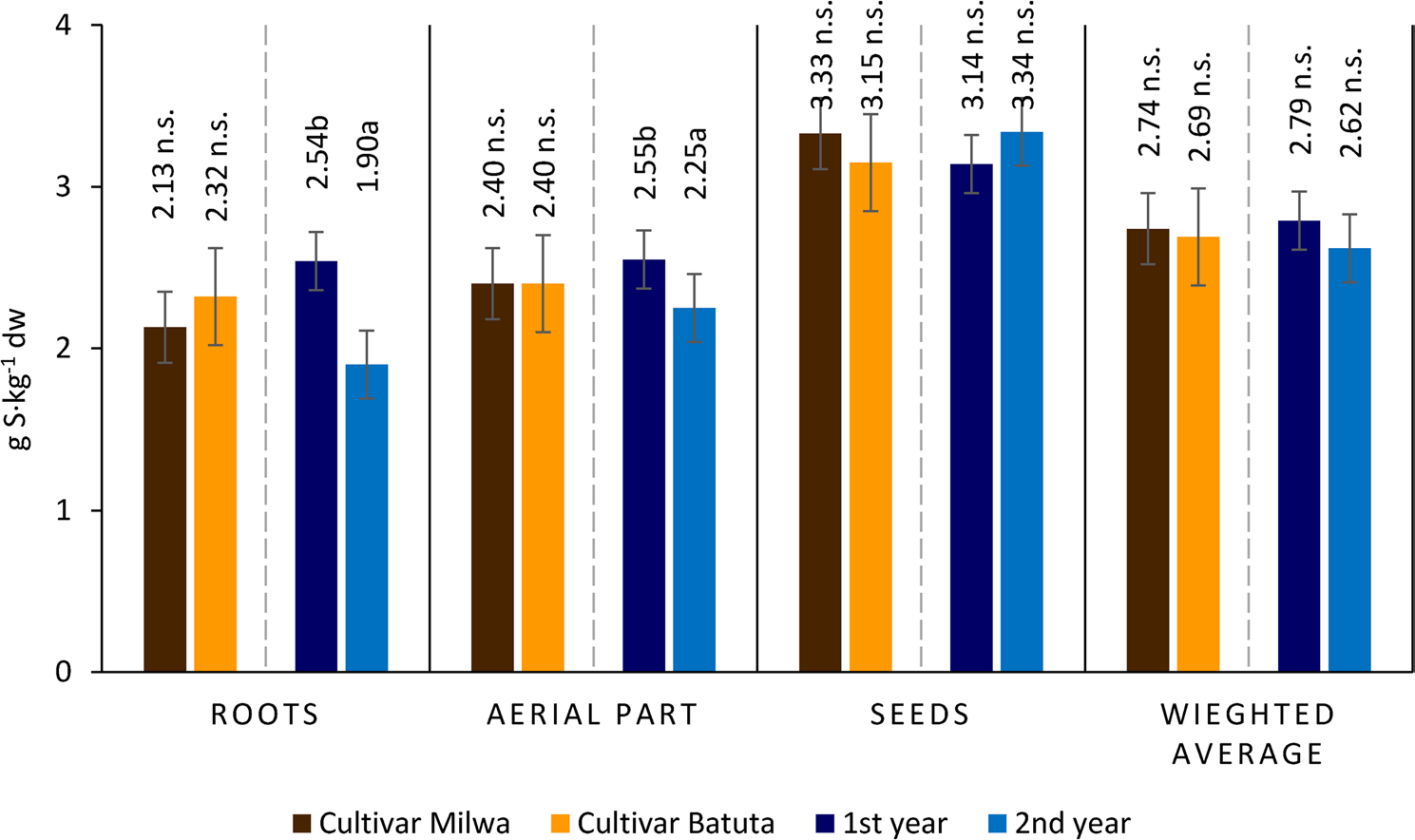
The content of sulphur in pea, mean values for cultivars and years in maturity growth stage, ± SD, g S·kg^− 1^ dw, LSD_0.05_ for years: roots 0.20, aerial part 0.23, n.s. – not significantly differ, a,b – means for investigated factors with different lowercase letters above the bars are significantly different

Sc did not depend significantly on the cultivar in any part of the plants (Fig. 1). Its content in the seeds and on average in the whole plant also did not vary significantly depending on the year. Sc in the roots and aerial part was higher in the conditions of the experiment conducted in the first year than in the second year.

### Nitrogen to sulphur ratio

The ratio of nitrogen content [45] to Sc (Table 5) in the separate parts of the plant and on average in the whole plant changed significantly during the stages of pea development (Table 6). The ratio in the aerial part and on average in the whole pea plants was highest in the first of the six stages. Subsequently, the N: S ratio on average in the whole plants was lower than in the four-leaf stage and did not change significantly. In the aerial part, the ratio decreased from the four-leaf stage to the first bud stage, and also from the 50% of pods stage to full maturity. In this part of the pea plant, the N: S ratio did not change significantly between the first bud stage and the 50% of pods stage. The N: S ratio in the roots changed according to the following pattern: four-leaf > three-internode < first bud = flowering < 50% of pods = maturity.

**Table 6:** Nitrogen to sulphur ratio in pea plant, mean values for varieties and years, ± SD, (g N·kg^− 1^ dw / g S·kg^− 1^ dw)

Parts of pea plants	Growth stages						LSD_0.05_	
	Four-Leaf	Three-internode	First bud	Flowering	50% Pods	Maturity		
Seeds						12.1 (± 1.0)	-	
Aerial part	15.9d (± 1.5)	11.7c (± 2.0)	9.2b (± 1.1)	8.6b (± 1.0)	9.1b (± 1.1)	5.3a (± 1.0)		1.2
Roots	7.5c (± 1.3)	5.1a (± 0.9)	6.4b (± 1.3)	6.2b (± 0.8)	8.5d (± 1.4)	8.6d (± 0.8)		1.0
For weighted averages of N and S content in whole pea plants	11.5b (± 0.9)	9.0a (± 0.9)	8.8a (± 1.1)	8.5a (± 1.0)	9.0a (± 1.1)	8.5a (± 0.9)		1.1

a, b,c, d – means for investigated factors with different lowercase letters in the rows are significantly different

**Fig. 2:**
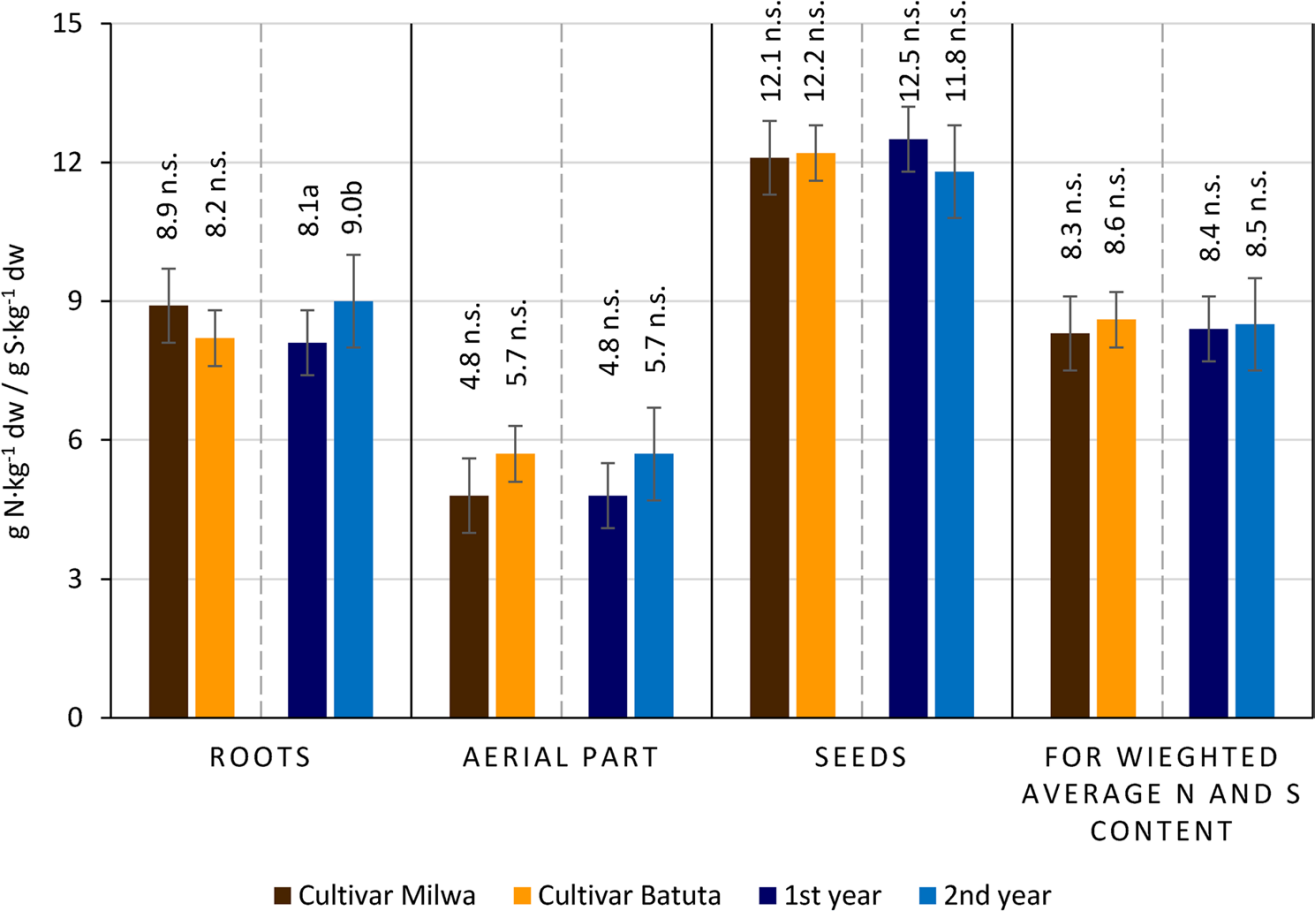
Nitrogen to sulphur ratio in pea plant, mean values for cultivars and years in maturity growth stage, ± SD, LSD_0.05_ for years: roots 0.9, n.s.– not significantly differ, a,b – means for investigated factors with different lowercase letters above the bars are significantly different

At full maturity, the N: S ratio in all separated parts and on average for the whole plant did not depend significantly on the cultivar or the year of the study (Fig. 2). An exception was its value for the roots in the second year, which was higher than in the first year.

### Sulphur uptake

**Fig. 3:**
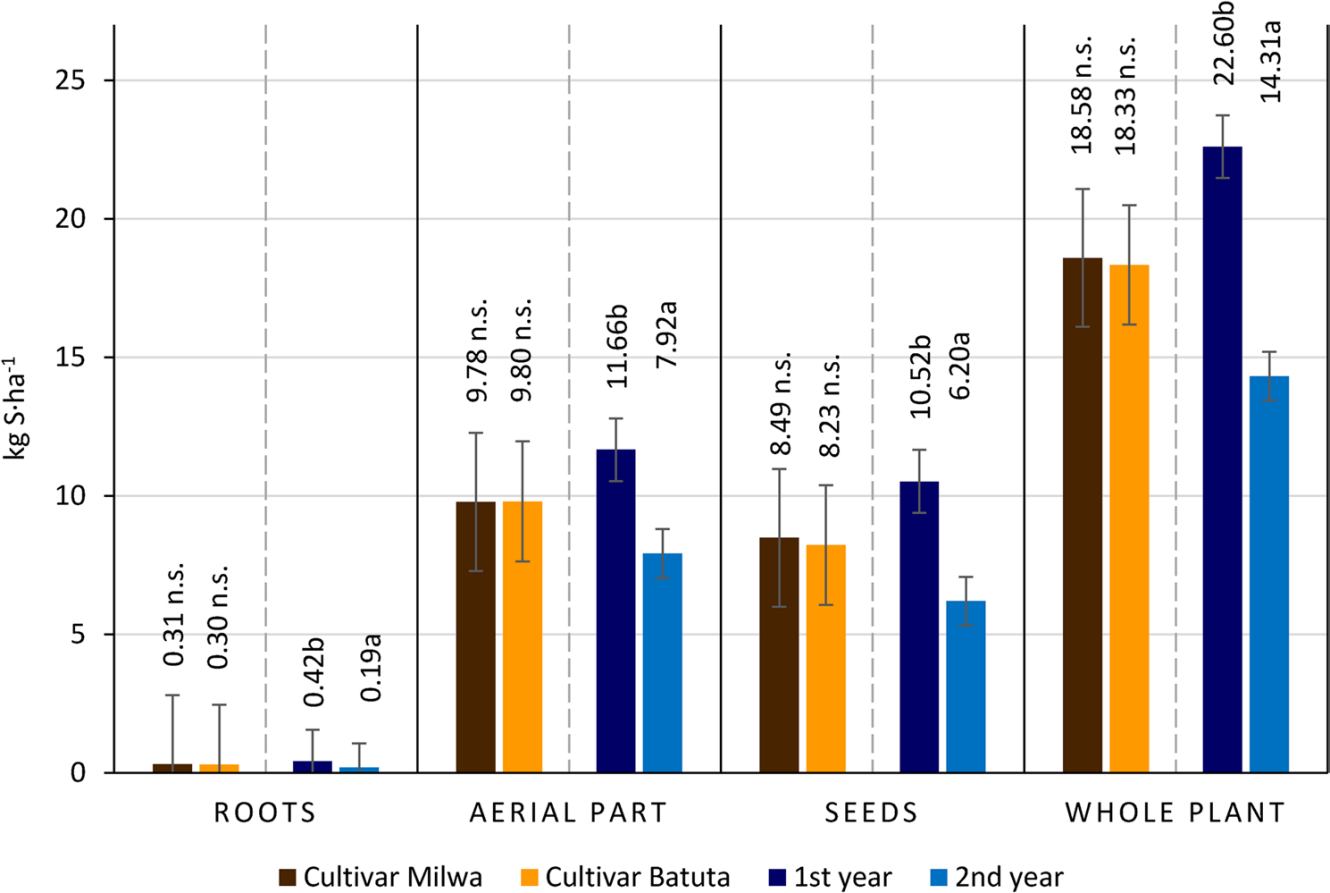
The uptake of sulphur by pea, mean values for cultivars and years in maturity growth stage, ± SD, kg S·ha^− 1^, LSD_0.05_ for years: roots 0.9, aerial part 2.26, seeds 1.62 and whole plant 2.82, n.s.– not significantly differ, a,b – means for investigated factors with different lowercase letters above the bars are significantly different

As in the case of Sc, Sup was significantly varied in successive stages of development (Table 7), and years of pea cultivation (Fig. 3). A significant increase in the amount of sulphur accumulated in the roots was obtained between the four-leaf stage and the three-internode stage of pea. The level of this macronutrient accumulated in the roots decreased in successive stages of development. The amount of sulphur accumulated in the aerial part of pea and its total uptake by whole plant increased from the four-leaf stage until the flowering stage. In later stages of development, the amount of sulphur accumulated in the entire pea plants did not change significantly. In the aerial part, the amount of sulphur accumulated at the flowering stage and at the 50% of pods stage did not differ significantly. In comparison with these stages, there was less sulphur accumulated in the aerial part at full maturity, because the seeds had been separated from it. When the amount of sulphur accumulated in the seeds was added to the amount accumulated in the aerial part without seeds, the statistical analysis showed that the amount of sulphur had increased significantly between the flowering stage and maturity. The calculations revealed that the increases in sulphur accumulation between the flowering stage and the 50% of pods stage, as well as between the 50% of pods stage and full maturity, were not significant. At full maturity of pea, the sulphur accumulated in the individual parts expressed as percentages of the total uptake by whole plant was as follows: roots 1.6%, aerial part 53.1%, and seeds 45.3%.

**Table 7:** The uptake of sulphur by pea, mean values for varieties and years, ± SD, (kg S·ha^− 1^)

Parts of pea plants	Growth stages						LSD_0.05_
	Four-Leaf	Three-internode	First bud	Flowering	50% Pods	Maturity	
Seeds						8.36 (± 2.38)	-
Aerial part	0.58a (± 0.11)	2.97b (± 0.57)	9.82c (± 2.94)	14.96d (± 5.04)	16.19d (± 4.58)	9.79c (± 2.33)	1.89
Aboveground part*	0.58a (± 0.11)	2.97b (± 0.57)	9.82c (± 2.94)	14.96d (± 5.04)	16.19de (± 4.58)	18.15e (± 4.39)	1.99
Roots	0.61b (± 0.09)	1.75d (± 0.61)	1.28c (± 0.55)	1.08c (± 0.33)	0.58b (± 0.18)	0.30a (± 0.13)	0.26
Whole plant	1.19a (± 0.16)	4.72b (± 0.75)	11.10c (± 3.30)	16.04d (± 5.33)	16.77d (± 4.69)	18.45d (± 4.49)	2.01

a, b,c, d – means for investigated factors with different lowercase letters in the rows are significantly different

* Addtitional statistical analysis made for sum for aerial part and seeds in maturity stage

Accumulation of sulphur in each separate part of the plant and its total uptake by the whole plant at full maturity did not depend significantly on the cultivar (Fig. 3). Pea grown in the conditions of the first year of the study accumulated more sulphur in the separated parts and in the whole plant than in the second year, by 121.0% in the roots, 47.2% in the aerial part, 69.7% in the seeds, and 57.9% in the whole plants.

### Internal efficiency index

The SIE value calculated for Sup by pea in the six development stages changed significantly in the separated parts and on average in the whole plant (Table 8). The value of this parameter for the roots, for the aerial part, and on average for the whole pea plant decreased from the four-leaf stage to the three-internode stage. Between the three-internode stage and first bud stage, it increased for these parts and on average in the whole plant. The SIE value for the roots and aerial part and on average for the pea whole plant also increased from the first bud stage until full maturity, but the significance of these differences were not confirmed statistically in in all cases. At full maturity, the values for this parameter can be ordered as follows: roots > aerial part > seeds.

**Table 8:** Sulphur Internal Efficiency index in pea plant, mean values for varieties and years, ± SD, kg^− 1^ dw per 1 kg Sup

Parts of pea plants	Growth stages						LSD_0.05_
	Four-Leaf	Three-internode	First bud	Flowering	50% Pods	Maturity	
Seeds						310 (± 22) 140* (± 17)	
Aerial part	323c (± 25)	253a (± 35)	284b (± 37)	333 cd (± 25)	362d (± 51)	423e (± 53)	30
Roots	250b (± 74)	131a (± 37)	218b (± 60)	249b (± 38)	383c (± 42)	462d (± 75)	33
Whole mass	285b (± 30)	205a (± 35)	275b (± 37)	327c (± 25)	362d (± 50)	372d (± 31)	24

a, b,c, d,e – means for investigated factors with different lowercase letters in the rows are significantly different

* The value obtained after dividing the seed yield by the amount of sulfur taken up by the whole plant

**Fig. 4:**
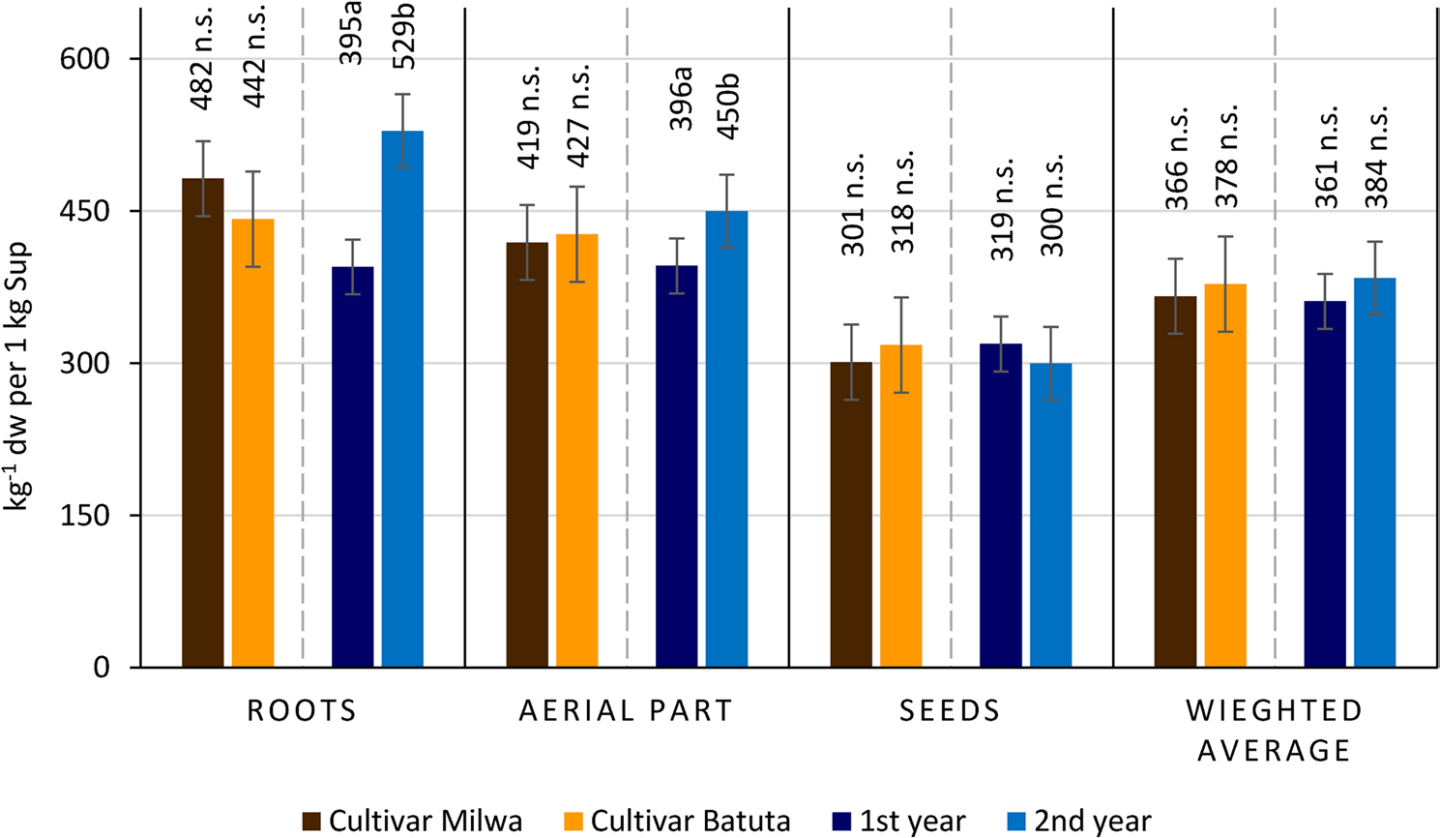
Sulphur Internal Efficiency index, mean values for cultivars and years in maturity growth stage, ± SD, kg^− 1^ dw per 1 kg Sup, LSD_0.05_ for years: roots 44, aerial part 45, n.s.– not significantly differ, a,b – means for investigated factors with different lowercase letters above the bars are significantly different

The SIE value of Sup by pea harvested at full maturity did not depend significantly on the cultivar for any part of the plant or on average for the whole plant (Fig. 4). Similarly, the value of this parameter for the seeds and on average for the whole biomass did not vary significantly depending on the year. SIE value for the roots and aerial part was greater in the conditions of the experiment conducted in the second year than in the first year.

### Sulphur harvest index

The SIE value calculated from the four-leaf stage to the 50% of pods stage for green matter and for the maturity stage for total sulphur accumulated in the aerial part and seeds significantly increased starting from the three-internode stage up to the 50% of pods stage (Table 9). The change between the 50% of pods stage and maturity was small and not significant.

**Table 9:** Sulphur Harvest Index for aerial part of pea plant (from four-leaf to 50% pods phase applies green mass, but in maturity phase applies aerial crop residues^1^ and seeds^2^) mean values for varieties and years, ± SD

Growth Stages						LSD_0.05_
Four-Leaf	Three-internode	First bud	Flowering	50% Pods	Maturity	
0.49a (± 0.05)	0.63b (± 0.10)	0.89c (± 0.03)	0.93d (± 0.01)	0.97e (± 0.01)	(0.53^1^+0.45^2^) 0.98e (± 0.05^1^, ± 0.05^2^, ± 0.05)	0.04

a, b,c, d,e – means for investigated factors with different lowercase letters in the rows are significantly different

**Fig. 5:**
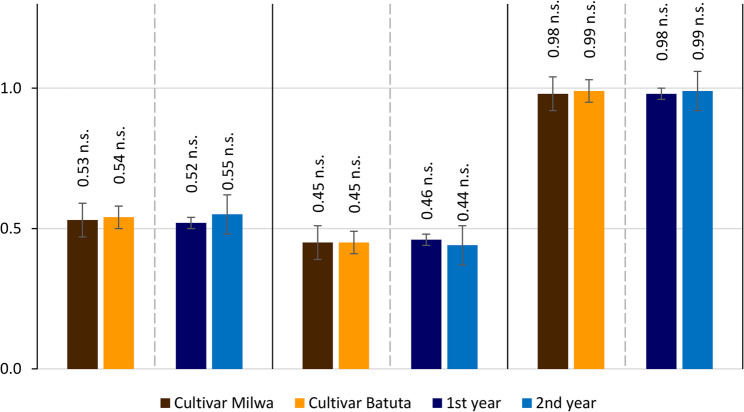
Sulphur Harvest Index, mean values for cultivars and years in maturity growth stage, ± SD, n.s.– not significantly differ

The SHI at full maturity of pea did not depend significantly on the cultivar or year of the study (Fig. 5).

## Discussion

Sulphur present in soil is taken up by plant roots in a metabolically neutral sulphate form (-SO_4_^2−^) [12, 36]. This is also the form in which this macronutrient is most often applied to the soil in agricultural practice [36, 46]. In the present study sulphur was applied to the soil in this form (-SO_4_^2−^), so it was immediately available in its entirety to the plant. The absorbed sulfur is loaded into the xylem and transported via transpiration to the shoot. In plants well supplied with sulfur, most of the sulfur is initially delivered to established leaves and then gradually redistributed, primarily to younger leaves, where it is assimilated into S amino acids [47]. In the fertilization method adopted in our study, the highest Sc in the roots and aerial part and on average for the whole pea plants was recorded at the initial growth stages. Despite high Sc in pea biomass in this stages, the amount accumulated in the tissues was low due to the low weight of the plants [45]. Correlation coefficients indicate that the amount of sulphur accumulated in the whole pea plants in the six development stages was correlated with the amount of biomass (r = + 0.973) and not with its content in the biomass (*r* = − 0.471). Thus, increased sulfur accumulation is closely linked to increased plant mass. High yields are possible with an adequate supply of sulfur to plants, because according to Li and coauthors [48], its deficiency leads to reduced plant metabolic activity, inhibition of the synthesis of enzymes involved in carbon metabolism, reduced photosynthesis rates, and increased accumulation of reactive oxygen species in plants. The own results indicate dynamic S uptake from the four-leaf stage to the flowering stage (during intensive biomass growth), as well as minor uptake from the flowering stage to maturity. Literature data indicate that pea requires sulphur availability throughout the growing season: first it is essential to the formation of root nodules, then in the nitrogen reduction process, and finally for the formation of sulphur-containing storage proteins [4, 13–15]. It is sensitive to a lack of available sulphur in the soil during the final development stages due to its poor distribution from the leaves to the seeds [4]. When sulphur supply is low, the plant’s transcriptional mechanism limits the synthesis of sulphur-rich proteins, reallocating available nitrogen to favor the production of sulphur-poor storage proteins that contain no or very few sulphur amino acids [49]. In the present study, the amount of sulphur accumulated in the roots systematically decreased beginning with the first bud stage up to full maturity. At the same time, the amount accumulated in the aerial part increased during this period. In addition, between the 50% of pods stage and full maturity, there was a marked decrease in the amount of sulphur accumulated in the aerial part without seeds (on average by 6.40 kg S·ha^− 1^, 39.5%). At full maturity, pea had accumulated 8.36 kg S·ha^− 1^ in the seeds. This data are indicative of uptake of sulphur from the soil as well as its remobilization from the vegetative organs to the seeds in the final stage of growth. Literature data also indicate that remobilization of sulphur is possible because protein degradation begins in the older leaves, after which protein amino acids are transformed into glutathione the main form of organic sulphur transported in the phloem as well as into S-methyl methionine, an important transporter of sulphur in legume plants [36]. Remobilization of sulphur is most intensive when its supply from the roots ceases in the seed-filling stage [4]. Thus the plant begins to mobilize resources from the leaves only when the roots are no longer able to meet the high demand during the formation of storage proteins in the seeds. The high rate of redistribution of sulphur obtained in the present study in the final growth stages of pea may be indicative of its limited availability from soil resources. These observations suggest that further experiments should be conducted to investigate the range of sulphur remobilization in conditions of varying levels of sulphur availability during the final development stages of pea. For example, these conditions can be created by dividing the total amount of sulphur to be applied in sulphate forms and applying it at selected development stages, by applying sulphur before sowing in forms which are slowly transformed into available forms for plants, or even by foliar application.

An increase in the amount of sulphur accumulated in the whole pea plants was accompanied by a decrease in its concentration in the biomass. The correlation coefficient between the mean Sc in the biomass of pea and the total weight of the plant was *r* = − 0.471, and the correlation coefficient between the mean Sc and the total Sup in the plant was *r* = − 0.611. This indicates the classic effect of dilution of the concentration in the increasing mass of the plant.

Sc below 2.0 g S·kg^− 1^ in the leaves is considered a deficiency, while concentrations above 30 g S·kg^− 1^ are considered toxic for various plants [36]. In the case of the aerial parts of pea, sulphur deficiency occurs at concentrations below 1.3–1.8 g S·kg^− 1^, while adequate content has been found to range from 2.0 to 6.0 g S·kg^− 1^, depending on the development stage [50]. Researchers obtained content of about 2.0 g S·kg^− 1^ in the leaves at the flowering stage and 1.8 g S·kg^− 1^ in the seeds when they applied 50 to 100 mg S per pot [24]. In that study, Sc in the shoots and roots up to 73 days of growth was within ranges of 2.8–2.3 and 10.8–7.4 g S·kg^− 1^, respectively, following application of 50 mg S per pot. In soybean, the thresholds for diagnosis of S deficiency have been determined to be (g S·kg^− 1^) 2.65 in the leaves and 2.06 in the shoots [36]. The pea grown in the own study contained from 4.06 to 2.40 g S·kg^− 1^ in the aerial part throughout the growing season, which indicates that it was well supplied with this macronutrient. This was also indicated by the Sc in the aerial part of pea up to the flowering stage, which remained above 3 g S·kg^− 1^. For comparison, Kolbe [33] obtained Sc of 2.74 g S·kg^− 1^ in young pea plants. In the seeds, we obtained on average 3.24 g S·kg^− 1^, which is also high in comparison to the findings of other authors, which range from 0.8 to 2.8 g S·kg^− 1^ [33, 51].

In a mathematical sense, the SIE index calculated in our experiment is the inverse of the content. This index presents how much biomass or usable yield is produced by the plant per unit of nutrient taken up [52]. In general, plants with high content of a nutrient will have lower values for this index than plants with lower content. A high SIE value indicates the production of a large amount of biomass with a low content of sulphur in the tissues, while a low value indicates ‘luxury uptake’, i.e. accumulation without conversion into biomass. We did not find values for SIE in pea during the entire growing period in the available literature. In our study, we obtained the lowest SIE values for whole weight calculated per 1 kg S accumulated in the three-internode stage (205) and the highest at maturity (372). In the final stage, the values for the seeds were 310 and 140 kg per 1 kg S accumulated in the seeds and per 1 kg S accumulated in the whole plant, respectively. If the whole plant takes up 8 kg sulphur on average (range 5–13) to produce one tonne of legume seeds [53], the mean SIE index is 125 (range 80–200). The value obtained in the own study (140) is within this range and is slightly higher than the average. This means that to produce one tonne of seeds, the pea plants took up slightly less than 8 kg S (7.1 kg S on average). High values for this index (close to 200) might have indicated that the plant was producing yield at low sulphur uptake, which could adversely affect the quality of the protein contained in it. Literature data indicate that in the experiments conducted, values of this index exceeding 200 were obtained for pigeon pea [54].

One of the most important synergistic effects in the plant is the interaction of sulphur and nitrogen. Without adequate sulphur supply, plants are unable to properly metabolize nitrogen. The ratio of N: S content describes their proportions. It is a useful parameter for diagnosing sulphur deficiency in legume plants [4]. Kolbe [33] calculated the N: S ratio for the content of these elements for young plants, straw, and grain of pea and reported values of 11.8, 10.5 and 20.2, respectively. In the present study, we obtained higher N: S ratios in the aerial part of pea only in the four-leaf stage (15.9), similar values in the three-internode stage (11.7), and lower values from the first bud stage to maturity (9.2–5.3) in comparison with the values given above for young plants and straw. Similarly, the value of this ratio in the seeds (12.1) was considerably lower than the value cited above. Pötzsch et al. [32] found that when sulphur content in the leaves at the flowering stage of pea was 2.9 g S·kg^− 1^ and the N: S ratio was 15.6, this was sufficient to meet the demand of pea for sulphur. All the values for this ratio obtained in the own study are optimal and low, indicating that pea was well supplied with sulphur. In the case of soybean, the critical N: S ratio, depending on the source, is above 16:1 [36] or above 17.5:1 [55] and indicates sulphur deficiency and difficulties with producing valuable protein.

To determine the efficiency of migration of sulphur from the vegetative parts (roots and aerial part) to the seeds, as well as from the roots to the above-ground parts of pea plants, we calculated the SHI index. This shows what portion of the sulphur taken up by the entire plant was stored in the usable part -usually the seeds. If the green matter is also useful as green fodder, it can also be used as a usable part and removed from the field. Therefore, in our study, we calculated the SHI index, which additionally reflects the proportion of sulfur accumulated in the aboveground part of the crop compared to total its uptake. The values for this index indicated in the literature, calculated at full maturity of pea, are 0.63 [32], for pigeonpea 0.27 [54]. In own study at full maturity of pea, the SHI for seeds was 0.45, and was within the specified range given above. This means that slightly less than half of the Sup by pea (45%) was located in the main yield (seeds) and removed from the field, while 55% could return to the soil in crop residue. When the green matter was treated as the main (usable) yield (from the four-leaf stage to the 50% of pods stage), the SHI values ranged from 0.49 (four-leaf stage) to 0.97 (50% of pods stage). When green matter of pea is harvested, which in agricultural practice usually takes place between the flowering stage and the 50% of pods stage, more than 90% (93–97%) of Sup is removed from the field, and only 3–7% remains in the soil together with the roots. With this method of managing the green mass of peas, post-harvest residues (roots) practically cannot be treated as a source of sulphur for the plant.

## Conclusions

In the adopted fertilization method, the Sc in the roots, above-ground parts, and in the whole mass was the highest in the initial stages of development (highest Sc in three-internode) and decreased until full maturity. Starting from the thre-inetrnode phase, changes in Sc did not cause changes in the average value of N: S ratio in the whole pea mass (it ranged from 8.5 to 9.0). Peas showed the highest rate of Sup in the phases of intensive mass accumulation, i.e. from the four-lef phase (total Sup 1.19 kg S⋅ha^− 1^) to the flowering phase (total Sup 16.04 kg S⋅ha^− 1^). In subsequent development phases of pea mainly redistributed this element from vegetative to generative organs (seeds). This may indicate a limited supply of sulphur from the root system during this period. At full maturity, the Sc in the above-ground part (2.40 g S⋅kg^− 1^ dw) and in the seeds (3.24 g S⋅kg^− 1^ dw), the N: S ratios (in this parts 8.5 and 12.1, respectively), and the SHI index for seeds (of 140) indicate the proper supply of peas with this macronutrient. Taking into account the whole, it is advisable to conduct further extended experiments in which, in addition to pre-sowing fertilization, sulphur would be applied in divided doses - presowing and top dressing (soil and even foliar application), and its utilisation coefficients from fertilizer would be additionally determined (they were not the aim of the current research). The increasing values of the SIE index in subsequent stages of pea development (from 0.49 to 0.98) indicate that the later the biomass is harvested, the greater part of the Sup is taken out of the field, and the smaller part remains in the soil along with the roots.

The examined pea parameters indicate that varieties for different purposes managed sulphur in a similar way.

Taking into account the sulfur management calculated for the wole mass of peas, the research conditions only differentiated Sup (as a result of yield differentiation), without significantly differentiating Sc, N:S ratio, and value of SIE and SHI indexes.

## Data Availability

The datasets used and analysed during the current study are available from the corresponding author on reasonable request.

## Abbreviations

BBCH scale: Biologische Bundesanstalt, Bundessortenamt und Chemische Industrie scale
CH_3_COOH: Acetic acid
COBORU: Polish National List of the Research Centre for Cultivar Testing
HCl: Hydrochloric acid
HNO_3_: Nitric acid
ICP:AES spectrometer: Inductively coupled plasma atomic emission spectroscopy
K: Potassium
KC1: Potassium chloride
LSD: Least significant difference
LV: Luvisols
(NH_4_)_2_SO_4_: Ammonium sulphate
n.s.: Not significantly differ
N: S ratio: Nitrogen to sulphur ratio
P: Phosphorus
Sc: Sulphur content
SD: Standard deviation
SHI: Sulphur harvest index
SIE: Sulphur internal efficiency index
Sup: Sulphur uptake
